# Estimated Plan Enrollment Outcomes After Changes to US Health Insurance Marketplace Automatic Renewal Rules

**DOI:** 10.1001/jamahealthforum.2021.1642

**Published:** 2021-07-16

**Authors:** David M. Anderson, Petra W. Rasmussen, Coleman Drake

**Affiliations:** 1 Duke University Margolis Center for Health Policy, Durham, North Carolina; 2 RAND Corporation, Santa Monica, California; 3 Graduate School of Public Health, University of Pittsburgh, Pittsburgh, Pennsylvania

## Abstract

**Importance:**

The American Rescue Plan increases premium subsidies for health insurance marketplace enrollees, potentially leading to situations in which enrollees could switch to other health care plans with lower premiums and less cost sharing (ie, deductibles and copayments). Current policy defaults enrollees to their current health care plan if they automatically renew their coverage, which may cause them to stay in health care plans that, because of the American Rescue Plan, are now dominated in that they have higher premiums and cost sharing than other options.

**Objective:**

To estimate the extent to which a smart default policy could reduce US health insurance marketplace enrollees’ cost sharing and premiums.

**Design, Setting, and Participants:**

Using 2018 individual enrollment data and 2021 premium data from California’s marketplace and the American Rescue Plan premium tax credit subsidy schedule, this economic analysis estimated the characteristics of enrollees’ default health care plans if they defaulted into 2021 health care plans under current and smart default policies. The analysis was conducted from March 20 to April 8, 2021.

**Main Outcomes and Measures:**

Characteristics of enrollees’ default health care plans under current and smart default policies, including net premiums, plan levels, and cost sharing.

**Results:**

The analytic sample consisted of 748 087 Covered California enrollees from 2018 (mean [SD] age, 44.80 [13.72] years; 408 410 [54.6%] women). Under current policy with the enhanced subsidies implemented under the American Rescue Plan, 5.8% of sample enrollees would default into dominated health plans. Of these enrollees, 98.0% would have incomes below 250% of the federal poverty level. A smart default policy would lead to a mean $102.47 decrease in monthly premiums (95% CI, $103.84-$101.10), a mean $1960 reduction in individual annual medical deductibles (95% CI, $1991-$1928), and a $49.56 reduction in specialty prescription copays (95% CI, $49.77-$49.34).

**Conclusions and Relevance:**

The findings of this economic analysis suggest that a smart default policy could avoid defaulting lower-income marketplace enrollees to objectively inferior health care insurance plans and may lead to large reductions in lower-income enrollees’ deductibles, copayments, and maximum out-of-pocket amounts. Implementation of a smart default policy could enable marketplace administrators to reduce the prevalence of underinsurance among lower-income marketplace enrollees.

## Key Points

**Question:**

Is a smart automatic renewal policy that defaults US health insurance marketplace enrollees to more generous health care plans with equivalent or lower premiums associated with enrollees’ health care costs?

**Findings:**

In this economic analysis of US marketplace health care plan choices under the American Rescue Plan, 5.8% of 748 087 California marketplace enrollees currently default to dominated health care plans with higher premiums and cost sharing; more than 98.0% of enrollees have low incomes. By comparison, an alternative smart default system would default many enrollees to more generous plans with approximately $100 lower monthly premiums and almost $2000 lower deductibles.

**Meaning:**

Policy makers should consider implementing smart defaults for future marketplace automatic reenrollment.

## Teaser

This economic analysis estimates the extent to which a smart default policy could reduce health insurance marketplace enrollees’ cost sharing and premiums.

## Introduction

More than 12 million people are currently insured through the health insurance marketplaces created by the Patient Protection and Affordable Care Act (ACA).^1^ As more enrollees depend on the marketplaces for health insurance in the long term, policies that affect year-over-year enrollment are increasingly important. Automatic reenrollment, which allows enrollees to stay insured between years without making active annual plan selection decisions, may be the most important of these policies. Automatic reenrollment typically defaults enrollees to the same plan in which they were enrolled in the previous year.

Allowing enrollees to automatically reenroll in their coverage presents trade-offs. One study^2^ found that automatic reenrollment increases year-over-year health insurance retention by 30 percentage points. A total of 3 945 010 (32.8%) of all enrollees during the 2021 open enrollment period were automatically reenrolled.^3^ Passive reenrollment into a default plan can cause returning enrollees to reenroll in a plan that has higher premiums and less generous benefits than other available health plans. This phenomenon, known as dominated health plan choice, is a common occurrence in private health insurance markets, including the health care marketplaces.^4,5,6,7,8^

The American Rescue Plan (ARP), signed into law in March 2021, increased premium tax credit subsidies through 2022. These subsidies reduce marketplace plans’ premiums for qualifying enrollees. For example, under the ACA, an individual earning 150% of the federal poverty level (FPL) would pay $50 per month for a benchmark silver plan, a standard marketplace plan. Under the ARP, the same individual would pay $0 for the same plan.^9^ The premium of the bronze plan, on the other hand, is not affected by the ARP subsidies because it was already priced at $0 after subsidies under the ACA. Although a price-sensitive, lower-income marketplace enrollee may prefer a zero-dollar premium, high-deductible bronze plan over a $50-monthly premium, low-deductible silver plan under the ACA subsidy regimen, the new ARP subsidies change this calculus by reducing the silver premium to zero. There is no reason to select the bronze plan over the silver plan with the ARP subsidies, because they have equivalent premiums and the silver plan requires less cost sharing—the bronze plan is dominated by the silver plan. The ARP increases premium tax credit subsidies relative to the ACA for all income levels (Table 1).

**Table 1.** Expected Income Contribution Percentages and Amounts for 2021 Under the Affordable Care Act (ACA) and American Rescue Plan (ARP) Subsidy Schedules^a^

Household income, % of federal poverty level	Expected income contribution, %^b^		Expected contribution amount, $^c^	
	ACA	ARP	ACA	ARP
100	2.07	0	22.01	0
133	3.10	0	43.84	0
150	4.14	0	66.03	0
200	6.52	2.00	138.66	42.53
250	8.33	4.00	221.44	106.33
300	9.83	6.00	313.58	191.40
350	9.83	7.25	365.84	269.82
400	9.83	8.50	418.10	361.53

^a^ Data from the Congressional Budget Office cost estimate of the reconciliation recommendations for the House Ways and Means Committee.

^b^ The expected income contribution percentage is the percentage of a modified adjusted gross income that a household must pay to purchase the benchmark silver plan available to them in their state’s marketplace, after applying premium tax credit subsidies. Premium tax credit subsidies cap the premium of the benchmark silver plan to ensure that the household’s premium is equal to its expected income contribution percentage. Premium tax credit subsidies may be applied to any marketplace plan, except catastrophic coverage.

^c^ The expected income contribution amount is the monthly amount (ie, monthly premium) a household must pay to purchase the benchmark silver plan available to them in their state’s marketplace, after applying premium tax credit subsidies.

The ARP’s increased premium tax credit subsidies may increase dominated health plan choice. Of particular concern are marketplace enrollees who selected low-premium but high–cost-sharing bronze plans in previous years. After premium tax credits are applied, zero-premium bronze plans are currently available to 42.0% of eligible marketplace enrollees; this percentage will increase under the ARP.^10,11,12^ Although bronze plans’ lower premiums are highly attractive to marketplace enrollees,^12^ bronze plans have higher deductibles, out-of-pocket maximums, and copayments than plans in other levels (ie, bronze, silver, gold, or platinum).^13^ However, if the enrollee does not log back into the marketplace to review how plans’ premiums have changed under the ARP, they may stick with the dominated bronze plan.

Dominated health care plan choice is especially concerning for lower-income marketplace enrollees, who are also eligible for cost-sharing reduction subsidies.^14^ These subsidies, which can only be applied to silver plans, reduce cost sharing (ie, deductibles, maximum out-of-pocket amounts, and copayments) such that silver plans offer far more financial protection than bronze plans for lower-income enrollees. For example, the bronze plan’s deductible is $6300, whereas the silver plan’s deductible is $150. However, lower-income marketplace enrollees are less likely to review changes in their health plan choices from year to year, instead relying on automatic reenrollment, and therefore are more likely to remain with their previous health care plan despite potentially large benefits to changing their plan.^15,16,17,18^

In this analysis, we used marketplace enrollment data from California’s marketplace to estimate how many enrollees will be defaulted to dominated health care plans under current automatic reenrollment policies with ARP subsidies. We propose an alternative smart default policy that would default enrollees to nondominated health care plans.^19^ We then estimate how the implementation of this policy would affect the affordability of and financial protection offered by marketplace health care plans. Last, we project how many enrollees would benefit from such a change in the 36 states using the HealthCare.gov marketplace.

## Methods

### Data and Sample

For this economic evaluation, our primary data sources were 2018 individual enrollment data and 2021 plan offering data from California’s health insurance marketplace, Covered California. Approximately 1.6 million people are insured with Covered California health plans.^20^ These data are particularly useful for identifying dominated health plan choices because California standardizes health plan cost sharing within a hierarchy of levels, meaning that cost sharing is always reduced as plan level increases (eg, silver plans always have lower deductibles than bronze plans).^21^ This study was deemed exempt from approval and informed consent by the University of Pittsburgh Institutional Review Board because the data are publicly available and deidentified. We followed the Consolidated Health Economic Evaluation Reporting Standards (CHEERS) reporting guideline.^22^

We imposed 3 sample restrictions. First, we limited the sample to Covered California enrollees who were enrolled in a Covered California plan in 2018 and were eligible to be defaulted to a health care plan in the following year. Second, we limited the sample to enrollees who received ACA premium subsidies, because unsubsidized enrollees did not face dominated health care plan choices. Third, we excluded American Indian/Alaska Native enrollees, because these enrollees have different cost-sharing subsidies than the rest of the population.^23^

We also used aggregate 2020 enrollment data from the Centers for Medicare & Medicaid Services to estimate the total number of marketplace enrollees on the federally facilitated marketplace, HealthCare.gov, who could be affected by dominated defaults without a smart default policy. These data report the total number of HealthCare.gov enrollees at the plan-level income band in 2020 for each state.^24^

### Dominated Health Care Plans and Smart Defaults

We considered an enrollee’s default health plan to be dominated when the enrollee could be defaulted to another health plan with more generous cost sharing without increasing their premium or changing their insurance company or provider network (group of health care practitioners) because these could be a key motivation for the enrollee’s initial choice of the plan (eAppendix in the Supplement).

We defined smart defaults as a default plan assignment algorithm that avoids defaulting enrollees to dominated health plans.^2,19^ Under current policy, enrollees are defaulted to their current plan in the following year.^2^ A smart default policy would determine whether an enrollee’s current plan would be a dominated health care plan in the following year. If the enrollee’s current plan would not be dominated in the following year, the enrollee would be defaulted to their current plan, as is the case under current policy. If the enrollee’s current plan would be dominated in the following year, the enrollee would be defaulted to the health care plan offered in their insurer’s provider network with the most generous cost sharing for the same or lower premium as their previous plan. For example, an enrollee covered by the bronze plan with a $0-monthly premium and a $6300 annual deductible would not be defaulted to the silver plan that, under ACA, had a $50-monthly premium and a $150 deductible. However, under ARP, the same silver plan has a $0 premium. Under the smart default policy with ARP subsidies, the enrollee would be defaulted to the silver plan because it has lower cost sharing for no higher premium than the bronze plan.

### Outcome Measures

Our outcomes were the characteristics of enrollees’ default health care plans. These attributes included premiums, plan levels, medical and prescription deductibles, out-of-pocket maximums, and copayments for primary and specialty care. We also reported the demographic characteristics of enrollees that would be affected by a smart default policy, including the plan level of the enrollee’s health care plan from the previous year, enrollee income as a percentage of the FPL, age, sex, and whether the individual enrolled with other household members.

### Statistical Analysis

We identified whether enrollees in our sample would be defaulted to a dominated health care plan, if they were defaulted to 2021 health plans, under 4 different policy regimens: (1) ACA premium tax credit subsidies with current default policy, (2) ACA premium tax credit subsidies with smart defaults, (3) ARP premium tax credit subsidies with current default policy, and (4) ARP premium tax credit subsidies with smart defaults. We did so by imputing each enrollee’s 2021 premium tax credit under the ACA and ARP subsidy schedules listed in Table 1. We based these calculations, as the marketplaces themselves do, on each enrollee’s age, household income, household size, and the marketplace health care plans available to them.^11,25^

We then identified enrollees whose default plans met the criteria to be dominated as described above. We applied smart defaults to enrollees in dominated plans under the ARP, assigning them new default health care plans per the smart default algorithm (ie, the plan from the same insurer with the same provider network that has the most generous cost sharing but would not result in a premium increase, after applying premium tax credits). We then calculated the statistical difference between the plan characteristics of these enrollees’ default plans under current default policy and smart default policy using 2-tailed *t* tests.

Last, we projected the number of HealthCare.gov enrollees who would be defaulted to dominated marketplace plans under the ARP without a smart default policy. We did so by multiplying the percentage of enrollees assigned to dominated default plans in our Covered California analysis by the number of HealthCare.gov enrollees. We adjusted for differences in FPL and plan level enrollment in California relative to the 36 states that use HealthCare.gov. For more details on this calculation, see eTable 1 in the Supplement. All analyses were conducted with Stata SE software, version 16.0 (StataCorp). A 2-sided *P* < .05 indicates statistical significance.

## Results

Our analytic sample consisted of 748 087 Covered California enrollees from 2018 (mean [SD] age, 44.80 [13.72] years; 408 410 [54.6%] women and 339 666 [45.4%] men). In 2018, 55.0% of returning enrollees in our sample automatically renewed their coverage. We estimated that, were these enrollees to be defaulted to the 2021 equivalents of their current plans with historical ACA subsidies, 24 417 (3.3%) of them would be defaulted to dominated health care plans. Under the augmented ARP subsidies, we estimated that 43 345 (5.8%) of the sample enrollees would be defaulted to dominated health care plans, an increase of 2.4 percentage points and a 77.5% increase in dominated default plan assignments.

Table 2 gives the demographic characteristics of the overall sample and those enrollees who would be defaulted to dominated health care plans with ACA and ARP subsidies. More than 98.0% of enrollees who would be defaulted to dominated health care plans had incomes less than 250% of the FPL—$32 200 for an individual and $66 250 for a family of 4^26^—under the ACA and the ARP subsidies. Under ACA subsidies, 91.7% of enrollees defaulted to dominated health care plans were enrolled in gold or platinum plans with comparatively low cost sharing. Under ARP subsidies, 43.8% of enrollees who would be defaulted to dominated health plans were enrolled in less generous bronze plans with relatively high deductibles, copayments, and maximum out-of-pocket amounts (ie, less generous cost sharing).

**Table 2.** Dominated Default Plan Assignments Under the Affordable Care Act (ACA) and the American Rescue Plan (ARP)^a^

Demographic characteristic	No. of enrollees (% of sample)		
	Overall (N = 748 087)	Enrollees defaulted to dominated health plans	
		ACA (n = 24 417)	ARP (n = 43 345)
FPL, %			
138-150	155 803 (20.8)	6570 (26.9)	11 533 (26.6)
>150-200	276 563 (37.0)	17 434 (71.4)	27 353 (63.1)
>200-250	152 262 (20.4)	326 (1.3)	3809 (8.8)
>250-400	163 459 (21.9)	87 (0.4)	650 (1.5)
Age, y			
0-17	16 351 (2.2)	138 (0.6)	172 (0.4)
18-34	236 376 (31.6)	9610 (39.4)	17 694 (40.8)
35-49	176 754 (23.6)	6333 (25.9)	11 104 (25.6)
≥50	318 606 (42.6)	8336 (34.1)	14 375 (33.2)
Sex			
Female	408 410 (54.6)	13 131 (53.8)	21 578 (49.8)
Male	339 666 (45.4)	11 285 (46.2)	21 765 (50.2)
Enrollment unit^b^			
Single	606 251 (81.0)	19 462 (79.7)	36 832 (85.0)
Family	141 836 (19.0)	4955 (20.3)	6513 (15.0)
Plan level^c^			
Bronze	206 868 (27.7)	1994 (8.2)	18 965 (43.8)
Silver no CSR	51 333 (6.9)	2 (0.0)	51 (0.1)
Silver CSR 73	63 231 (8.5)	24 (0.1)	543 (1.3)
Silver CSR 87	194 183 (26.0)	6 (0.0)	1357 (3.1)
Silver CSR 94	131 554 (17.6)	0	0
Gold	76 176 (10.2)	20 150 (82.5)	20 188 (46.6)
Platinum	24 742 (3.3)	2241 (9.2)	2241 (5.2)

Abbreviations: CSR, cost-sharing reduction; FPL, federal poverty level.

^a^ Under the current policy, households are defaulted to their health care plan from the previous year. A health care plan is dominated if there is another within-network health plan available that has the same or a lower premium and is of a higher plan level (ie, lower deductibles and copayments). Data are from 2018 Covered California administrative enrollment data and 2021 Covered California premiums.

^b^ An enrollment unit of single means the enrollee is covered by themselves. An enrollment unit of family means 2 or more family members are covered under the same health care plan.

^c^ Cost-sharing reduction subsidies reduce cost sharing and increase actuarial value for households that earn between 100% and 250% of the FPL who purchase a silver plan in 3 tiers: households earning 100% to 150% of the FPL qualify for 94% actuarial value plans (Silver CSR 94), households earning 151% to 200% of the FPL qualify for 87% actuarial value plans (Silver CSR 87), and households earning 201% to 250% of the FPL qualify for 73% actuarial value plans (Silver CSR 73). Standard silver plans without CSR benefits have 70% actuarial value and are available to households with incomes above 250% of the FPL.

The Figure illustrates how the default plan levels of enrollees in dominated health care plans would change by transitioning from current default policy to smart default policy (see eTable 2 in the Supplement for the number of enrollees moving plan tiers under the smart default policy). Under current default policy with ARP subsidies, 43.8% of enrollees in dominated plans would be defaulted to dominated bronze plans with 60% actuarial value. Under the smart default policy, 79.2% of them would be defaulted to significantly more generous silver cost-sharing reduction plans with 87% or 94% actuarial value. In addition, under the current policy, 46.6% of enrollees in dominated plans would be defaulted to dominated gold plans. With smart defaults, they would have their deductibles, copayments, and maximum out-of-pocket amounts reduced by being defaulted to silver plans with cost-sharing reduction subsidies.

**Figure.**
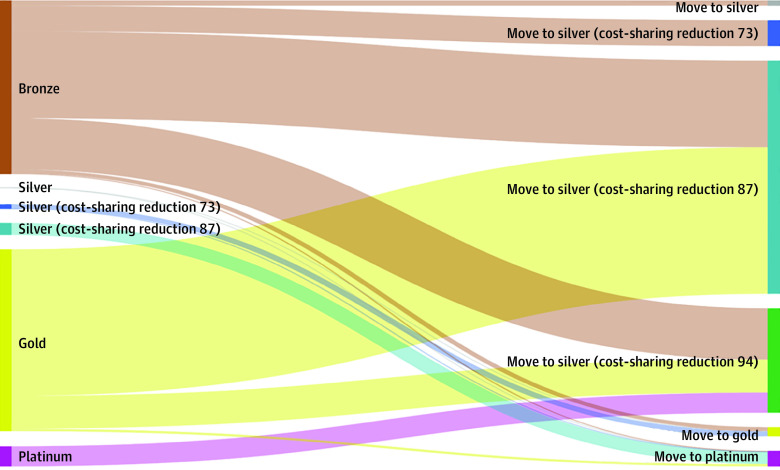
Changes in Actuarial Value of Default Health Plans Under the American Rescue Plan With Smart Defaults Data are from 2018 Covered California administrative enrollment data and 2021 Covered California premiums. Sample consists of 43 345 Covered California enrollees who would be assigned to dominated default health plans in 2021 under the American Rescue Plan, per the simulation discussed in the Methods section. The left side of the figure shows the plan levels of sample enrollees' default plans under current default policy. The right side of the figure shows the plan levels of sample enrollees' default plans under smart default policy. In all cases, the smart default policy defaults sample enrollees to more generous plan levels without increasing their premiums.

Table 3 indicates how smart defaults would change default plan characteristics for enrollees defaulted to dominated plans under the ARP, relative to current default policy. The percentage of affected enrollees with a $1 premium would increase from 52.9% to 68.4% under the smart default policy. These $1 premiums would be $0 in most other states; California has a premium floor of $1, resulting from a mandate that its marketplace insurers provide abortion coverage.^11,27^ Among affected enrollees with a net premium higher than $1, mean monthly premiums would decrease by $102.47 (95% CI, $101.10-$103.84), from $148.03 to $76.85. Single enrollees would see a mean $1960 reduction in medical deductibles (95% CI, −$1991 to −$1928), a mean $164.51 reduction in prescription deductibles (95% CI, −$166.99 to −$162.03), and a mean $4978 reduction in maximum out-of-pocket amounts (95% CI, −$5000 to −$4956). Smart defaults would also effect a mean $32 reduction in primary care copayments (95% CI, −$32.17 to −$31.84) and a mean $49.56 reduction in specialist copayments (95% CI, −$49.77 to −$49.34), an approximately two-thirds reduction in copayments compared with baselines of $46.47 for primary care copayments and $75.21 for specialist copayments.

**Table 3.** Changes in Default Health Care Plan Characteristics Under Smart Default Policy^a^

Plan characteristic	Mean (n = 43 345 enrollees)^b^			
	Current default policy	Smart default policy	Difference (95% CI)^c^	*P* value
Default monthly premiums^d^				
Premium of $1, %	52.9	68.4	15.6 (15.2 to 15.9)	<.001
Premium, $	70.36	25.06	−45.31 (−45.99 to −44.62)	<.001
Premium if>$1, $	148.03	76.85	−102.47 (−103.84 to −101.10)	<.001
Plan level, %^e^				
Bronze	43.8	0.0	−43.8 (−44.2 to −43.3)	<.001
Silver no CSR	0.1	1.3	1.2 (1.1 to 1.4)	<.001
Silver CSR 73	1.3	6.5	5.3 (5.0 to 5.6)	<.001
Silver CSR 87	3.1	59.4	56.3 (55.8 to 56.8)	<.001
Silver CSR 94	0.0	26.4	26.4 (26.0 to 26.8)	<.001
Gold	46.6	2.3	−44.3 (−44.8 to −43.8)	<.001
Platinum	5.2	4.0	−1.1 (−1.4 to −0.9)	<.001
Deductible for medical, $^f^				
Single	3096	1136	−1960 (−1991 to −1928)	<.001
Family	2938	2423	−515 (−634 to −395)	<.001
Deductible for prescription, $^f^				
Single	245.11	80.59	−164.51 (−166.99 to −162.03)	<.001
Family	231.87	172.57	−59.30 (−68.74 to −49.86)	<.001
Maximum out-of-pocket cost, $^f^				
Single	7828	2850	−4978 (−5000 to −4956)	<.001
Family	15 547	5866	−9681 (−9781 to −9580)	<.001
Copayment, $				
Primary care	46.47	14.47	−32.00 (−32.17 to −31.84)	<.001
Specialist	75.21	25.65	−49.56 (−49.77 to −49.34)	<.001

Abbreviation: CSR, cost-sharing reduction.

^a^ Data are from 2018 Covered California administrative enrollment data and 2021 Covered California premiums.

^b^ The enrollment unit of 36 832 enrollees was single (ie, 1 enrollee per health plan); the enrollment unit of 3014 enrollees was family (ie, >1 enrollee per health plan).

^c^ Differences are calculated using bivariate 2-tailed *t* tests.

^d^ Premiums are reported as the monthly premium of the enrollee’s default plan, net of subsidies. The first row reports the percentage of enrollees with a default monthly premium equal to $1 per person. The second row reports mean default monthly premiums. The third row reports mean default monthly premiums, conditional on premiums being greater than $1 per person.

^e^ Cost-sharing reduction subsidies reduce cost sharing and increase actuarial value for households that earn 100% to 250% of the federal poverty level who purchase a silver plan in 3 tiers: households earning 100% to 150% of the federal poverty level qualify for 94% actuarial value plans (Silver CSR 94), households earning 151% to 200% of the federal poverty level qualify for 87% actuarial value plans (Silver CSR 87), and households earning 201% to 250% of the federal poverty level qualify for 73% actuarial value plans (Silver CSR 73). Standard Silver plans without CSR benefits have 70% actuarial value and are available to households with incomes above 250% of the federal poverty level.

^f^ Medical deductibles, prescription deductibles, and maximum out-of-pocket amounts all differ, depending on whether the enrollment unit is single (1 enrollee) or family (2 or more enrollees). These plan characteristics are reported for the subsamples who experience them (eg, single medical deductibles are reported for the 36 832 single enrollees and family medical deductibles are reported for the 3014 family enrollees).

We projected that approximately 327 000 enrollees in the 36 states using the HealthCare.gov marketplace, or approximately 4.4% of subsidized enrollees, will be defaulted to dominated health care plans under the current policy. Our projections indicate that approximately 319 000 (97.0%) of these enrollees have incomes below 250% of the FPL, and 230 000 (70.0%) are enrolled in bronze plans (eTable 1 in the Supplement).

## Discussion

This economic evaluation of dominated marketplace health care plan choice under the ARP estimates that the percentage of enrollees defaulted to dominated health care plans will increase by nearly 80% because of the changes in premium tax credits from the ARP, potentially affecting more than 327 000 marketplace enrollees nationwide. Nearly half of the affected enrollees in our analysis were enrolled in bronze plans, suggesting that a smart default policy could switch many of these overwhelmingly lower-income enrollees from bronze plans with high deductibles to silver plans with cost-sharing reduction subsidies that drastically reduce and, in some cases, even eliminate deductibles and copayments. A smart default policy would also reduce the mean premium paid by these households by approximately $102 per month.

Automatic reenrollment is essential for maintaining health insurance enrollment because it allows people to stay insured if they simply continue to pay their premium. However, not having to pay attention to yearly changes in health care plan offerings can cause enrollees to overlook plans with lower premiums and more generous benefits.^19^ Prior research^18,28,29^ has found that the psychological and time costs required to search for a new health care plan are particularly costly to individuals with low socioeconomic status. This finding is particularly concerning for dominated health care plan choice in the marketplaces because this analysis found that nearly all the enrollees who will default to dominated health care plans under the ARP have incomes under 250% of the FPL.

The current policy that defaults lower-income enrollees to dominated bronze plans with high deductibles is likely to underinsure marketplace enrollees.^24^ A smart default policy would switch lower-income enrollees from bronze to silver plans with cost-sharing reduction subsidies. Reducing deductibles and copayments in this manner would likely lead to better health outcomes. For example, high deductibles and copayments have been shown to delay or prevent patients from filling prescriptions for cancer, diabetes, and other life-threatening conditions.^30,31,32,33^ By removing lower-income marketplace enrollees from bronze plans, which are similar to high-deductible health care plans, smart defaults could reduce inequities in health care use experienced by lower-income populations.^34^ In addition, reducing deductibles and out-of-pocket maximums can offer protection against bankruptcy for patients with chronic health conditions.^35^

### Limitations

This study has 3 main limitations. First, the analysis is limited to Covered California’s 2018 enrollment data. However, Covered California is the largest marketplace in the US, and its characteristics likely lead to conservative estimates of the effects of dominated defaults for 2 reasons. California standardizes deductibles and copayments across plan levels and limits the number of plans insurers may offer, which may make plan selection easier and thereby reduce dominated health care plan enrollment. California’s robust marketplace outreach also may reduce dominated health plan enrollment—the 36 states using the HealthCare.gov platform have had limited enrollee outreach since 2017.^7,36,37^

A second limitation is that 2022 premium data are not yet available. However, marketplace premiums have not changed significantly during the last several years, even in 2021 after the COVID pandemic.^38^ Should this trend continue, use of 2022 premium data rather than 2021 premium data would have a negligible effect on this analysis.

A third limitation is the possibility that enrollees may be more likely to switch health care plans in response to the passage of the ARP.^39^ Such switching could diminish the importance of smart defaults. However, previous analyses^7,17,40,41,42^ have found that enrollees, particularly lower-income ones, are not highly responsive to changes in year-over-year premiums or responsive to advertising or nudges.

## Conclusions

The findings of this economic analysis suggest that the US federal and state marketplaces should consider implementing smart defaults for future health insurance marketplace automatic reenrollment. A smart default policy avoids defaulting lower-income marketplace enrollees to objectively inferior health care insurance plans. In so doing, a smart default policy may lead to large reductions in lower-income enrollees’ deductibles, copayments, and maximum out-of-pocket amounts. Smart defaults may reduce underinsurance for hundreds of thousands of lower-income Americans, potentially enabling access to lifesaving medical care, minimizing cost barriers to accessing health care, and reducing the probability of health care–related bankruptcy. Premiums would also be reduced and, in some cases, eliminated. Although marketplace enrollees are free to opt out of their default plans, more than 80% of them do not. Smart defaults are therefore a powerful tool for policy makers to shape health insurance plan selection. In conclusion, implementation of a smart default policy would enable marketplace administrators to reduce the prevalence of underinsurance among lower-income marketplace enrollees.

## Notes

Supplement. **eAppendix.** Matching Similar Plans **eTable 1.** Estimated Dominated Health Plan Default Assignments for HealthCare.gov Enrollees Under the American Rescue Plan **eTable 2.** Plan Tier Movement for Covered California Enrollees Under Smart Default Policy
