# Supplementary material for: Estimated Plan Enrollment Outcomes After Changes to US Health Insurance Marketplace Automatic Renewal Rules
Source: JAMA Health Forum. 2021 Jul 16;2(7):e211642. doi: 10.1001/jamahealthforum.2021.1642 (PMC8796975; doi:10.1001/jamahealthforum.2021.1642)
Supplement: Supplement. — eAppendix. Matching Similar Plans eTable 1. Estimated Dominated Health Plan Default Assignments for HealthCare.gov Enrollees Under the American Rescue Plan eTable 2. Plan Tier Movement for Covered California Enrollees Under Smart Default Policy [file jamahealthforum-e211642-s001.pdf]

Supplementary Online Content

Anderson DM, Rasmussen PW, Drake C. Estimated plan enrollment outcomes after changes to US health insurance marketplace automatic renewal rules. *JAMA Health Forum.* 2021;2(7):e211642. doi:10.1001/jamahealthforum.2021.1642

eAppendix. Matching Similar Plans

eTable 1. Estimated Dominated Health Plan Default Assignments for HealthCare.gov Enrollees Under the American Rescue Plan

eTable 2. Plan Tier Movement for Covered California Enrollees Under Smart Default Policy

This supplementary material has been provided by the authors to give readers additional information about their work.

© 2021 Anderson D et al. *JAMA Health Forum*.

eAppendix. Matching Similar Plans

All insurance plans sold on both state-based marketplaces like Covered California and the federal marketplace, Healthcare.gov have common identifiers that we leveraged to identify similar plans for the purposes of the domination analysis. The plan that a consumer chose had the following distinct data elements:

- 5 character HIOS ID which identifies the insurance company
- Provider Network ID
- Plan Type (HMO, PPO, EPO, POS)
- Metal Level

The unique plan ID, a 16-character string consisting of the HIOS ID, the state abbreviation, a unique identifier substring and a two-character plan variant identifier defines the characteristics of the plan a enrollee purchased. A similar plan to the plan that a enrollee had purchased is defined as having the same HIOS ID, Provider Network ID and Plan Type. A similar plan will vary only in metal level. Operationalizing this process on Healthcare.gov would use the same data elements with the potential to add a formulary identifier to match on prescription drug availability for similar plans.

The gross premium of a enrollees' plan is the sum of the individual premiums rated by age within a rating area. Each enrollee is expected to pay an amount of monthly premium for the benchmark plan, which is the second least expensive silver plan. The expected contribution is a function of enrollee income as a percentage of the federal

© 2021 Anderson D et al. *JAMA Health Forum*.

poverty level. Lower income enrollee have lower expected contributions. The federal subsidy is the difference between the benchmark premium and the expected contribution. The expected contribution amounts were significantly lowered in the American Rescue Plan.   The net premium is what the enrollee pays after the application of federal premium subsidies. If a enrollee chooses a plan that is priced below the benchmark plan, their net premium decreased, bounded by zero. If a enrollee chooses a plan that is priced above the benchmark plan, the enrollee pays the full incremental premium above the benchmark plan.

A second set of subsidies are available to enrollee earning between 100-250% FPL. These Cost-Sharing Reduction (CSR) subsidies are intended to reduce out of pocket spending by lowering deductibles, co-pays, coinsurance and maximum out of pocket limits. CSR plans are only available to enrollee that purchase silver plans. Enrollee earning between 100-150% FPL qualify for "high" CSR, enrollee earning over 150% to 200% FPL qualify for "medium" CSR while enrollee earning between 200% to 250% FPL qualify for "low" CSR plans. High CSR silver plans have less cost sharing than any other plan, while medium CSR silver plans have less cost sharing then gold plans.

© 2021 Anderson D et al. *JAMA Health Forum*.

eTable 1. Estimated Dominated Health Plan Default Assignments for HealthCare.gov Enrollees Under the American Rescue Plan[a]			
**Total HealthCare.gov Enrollees with Premium Tax Credits by Income and Metal Levels**	**2020 HealthCare.gov Enrollees with Premium Tax Credits**	**Covered California Enrollees Assigned to Dominated Default Health Plans (%)**	**National estimate**
**100-150% FPL**			
Bronze	366,955	33.94%	124,555
Silver	2,629,713	0.01%	315
Gold	31,453	100%	31,453
Platinum	2,560	100%	2,560
**>150-200% FPL**			
Bronze	405,487	16.13%	65,389
Silver	1,234,562	0.70%	8,667
Gold	49,676	100%	49,676
Platinum	3,954	0%	0
**>200-250% FPL**			
Bronze	565,682	5.66%	32,028
Silver	473,392	0.86%	4,050
Gold	150,915	0.15%	227
Platinum	5,550	0%	0
**>250-300% FPL**			
Bronze	436,180	1.25%	5,453
Silver	161,325	0.13%	216
Gold	117,927	0%	0
Platinum	5,482	0%	0
**>300-400% FPL**			
Bronze	516,258	0.40%	2,083
Silver	160,531	0.01%	21
Gold	135,640	0%	0
Platinum	8,302	0%	0
**Total**	**7,461,544**	**-**	**326,694**
[a] Data are from 2018 Covered California administrative enrollment data, 2021 Covered California premiums, and 2020 HealthCare.gov enrollment data.			

© 2021 Anderson D et al. *JAMA Health Forum.*

eTable 2. Plan Tier Movement for Covered California Enrollees Under Smart Default Policy[a]

Baseline metal tier	Smart default metal tier (n)						
	**Bronze**	**Silver**	**Silver 73**	**Silver 87**	**Silver 94**	**Gold**	**Platinum**
Bronze	-	585	2,836	9,487	5,533	416	108
Silver	-	-	-	-	-	50	1
Silver 73	-	-	-	-	-	531	12
Silver 87	-	-	-	-	-	-	1,357
Silver 94	-	-	-	-	-	-	-
Gold	-	-	-	16,263	3,657	-	268
Platinum	-	-	-	-	2,241	-	-

[a] Data are from 2018 Covered California administrative enrollment data and 2021 Covered California premiums.

Sample consists of 43,345 Covered California enrollees that would be assigned to dominated default health plans in 2021 under American Rescue Plan, per the simulation discussed in the methods section.

© 2021 Anderson D et al. *JAMA Health Forum*.
